# Auditory stream segregation of amplitude-modulated narrowband noise in cochlear implant users and individuals with normal hearing

**DOI:** 10.3389/fpsyg.2022.927854

**Published:** 2022-09-02

**Authors:** Alexandria F. Matz, Yingjiu Nie, Harley J. Wheeler

**Affiliations:** ^1^Department of Otolaryngology, Eastern Virginia Medical School, Norfolk, VA, United States; ^2^Department of Communication Sciences and Disorders, James Madison University, Harrisonburg, VA, United States; ^3^Department of Speech-Language-Hearing Sciences, University of Minnesota, Twin Cities, Minneapolis, MN, United States

**Keywords:** auditory stream segregation, cochlear implants, narrowband noise, spectral separation, amplitude modulation, build-up effect

## Abstract

Voluntary stream segregation was investigated in cochlear implant (CI) users and normal-hearing (NH) listeners using a segregation-promoting objective approach which evaluated the role of spectral and amplitude-modulation (AM) rate separations on stream segregation and its build-up. Sequences of 9 or 3 pairs of A and B narrowband noise (NBN) bursts were presented which differed in either center frequency of the noise band, the AM-rate, or both. In some sequences (delayed sequences), the last B burst was delayed by 35 ms from their otherwise-steady temporal position. In the other sequences (no-delay sequences), the last B bursts were temporally advanced from 0 to 10 ms. A single interval yes/no procedure was utilized to measure participants’ sensitivity ( $d^{\prime }$ ) in identifying delayed vs. no-delay sequences. A higher $d^{\prime }$ value showed the higher ability to segregate the A and B subsequences. For NH listeners, performance improved with each spectral separation. However, for CI users, performance was only significantly better for the condition with the largest spectral separation. Additionally, performance was significantly poorer for the largest AM-rate separation than for the condition with no AM-rate separation for both groups. The significant effect of sequence duration in both groups indicated that listeners made more improvement with lengthening the duration of stimulus sequences, supporting the build-up effect. The results of this study suggest that CI users are less able than NH listeners to segregate NBN bursts into different auditory streams when they are moderately separated in the spectral domain. Contrary to our hypothesis, our results indicate that AM-rate separation may interfere with the segregation of streams of NBN. Additionally, our results add evidence to the literature that CI users build up stream segregation at a rate comparable to NH listeners, when the inter-stream spectral separations are adequately large.

## Introduction

Auditory stream segregation (also known as auditory streaming) refers to the process that allows listeners to interpret multiple sounds coming from different sources and assign those sounds to individual sound generators (Moore and Gockel, 2012). For example, normal-hearing (NH) listeners use stream segregation abilities to separate a talker at a noisy party or isolating the violin among the other instruments in an orchestra (Bregman, 1990, Chapter 1). Stream segregation has been shown to be related to the degree of the perceptual differences across sound streams (Moore and Gockel, 2002, 2012) in various domains such as frequency (e.g., Bregman and Campbell, 1971; Warren and Obusek, 1972; Dannenbring and Bregman, 1976), amplitude-modulation rate (AM-rate; e.g., Grimault et al., 2002; Nie and Nelson, 2015), pitch (e.g., Assmann and Summerfield, 1987; de Cheveigné, 1997), etc. When the acoustical inter-stream differences are adequately prominent, NH listeners may perceive separated auditory streams without voluntarily directing their attention to segregating the streams (e.g., van Noorden, 1975). This process is referred to as obligatory segregation and is generally noted to be driven by the stimulus (Bregman, 1990, Chapter 4). Conversely, when these differences are indistinct, listeners will experience obligatory integration, where they perceive only one auditory stream even when attempting to segregate signals into different streams (Bregman, 1990, Chapter 4). When the salience of these differences is ambiguous for the obligatory processing, NH listeners can intentionally direct their attention to perceptually separating or integrating auditory streams (van Noorden, 1975; Bregman, 1990, Chapter 4). These top-down processes are referred to as voluntary segregation and voluntary integration, respectively (Bregman, 1990, Chapter 4).

In everyday life, listeners are in complex auditory scenes where the differences between concurrent signals are often ambiguous, such as conversing in a restaurant or a cocktail party with varying background noises. Therefore, voluntary segregation is frequently employed by listeners to differentiate the interested auditory stream from interferences. Presumably, cochlear implants (CI) users need to engage in voluntary segregation in more listening conditions than do NH listeners, as the degraded auditory cues from cochlear implants may result in increased ambiguity of the differences between auditory streams (e.g., Fu and Nogaki, 2005). Despite such frequent adoption of voluntary stream segregation by CI users, this process remains poorly understood in many aspects. Particularly, the literature is lacking in comparisons between CI users and NH listeners in their ability to voluntarily segregate sound sequences based on the same inter-sequence acoustical differences. Findings of such comparisons may improve the understanding of the cues CI users utilize for sequential segregation with the facilitation of focused attention, which is relevant to speech perception in noise in their daily life (Nogueira and Dolhopiatenko, 2022). The current study was aimed to fill in this gap by comparing the two groups with manipulations of three acoustic attributes of the sound sequences—frequency, amplitude-modulation rate (referred to as AM-rate hereafter), and duration of the sequence.

In laboratory research, auditory stream segregation has been assessed using both subjective and objective paradigms. In subjective paradigms, listeners report their perception of the number of sound streams perceived. In objective paradigms, stream segregation is indexed by behavioral performance in the purportedly-designed listening tasks that can assess either voluntary or obligatory stream segregation. To assess voluntary segregation, the listening tasks may be arranged to be segregation-facilitating, such that they presumably require listeners to make effort to segregate auditory streams to achieve better performance. Thus, better performance in the segregation-facilitating objective paradigms indexes stronger voluntary segregation. To assess obligatory segregation, the listening tasks may be arranged to be integration-facilitating, in other words, segregation-hindering, such that listeners are awarded with better task performance for their mental effort made to integrate the auditory streams. Specifically, better performance in the integration-facilitating objective paradigms indexes stronger obligatory segregation.

One of the drawbacks with using subjective paradigms is the subject bias, such as listeners adopting different perceptual criteria for reporting stream segregation. For CI users, this bias may be partly attributed to the uncertainty of their discernment of auditory streams. CI users are provided signals by way of electrical stimulations with degraded auditory cues, unlike NH listeners. As a result, it is unclear whether CI users comprehend the concept of auditory streams consistently both within the group and when compared with NH listeners. Hence, the subjective reports of stream segregation may not be based on the same perception between CI users and NH listeners as well as among CI users. In contrast, with an objective paradigm, listeners are not required to comprehend the concept of auditory streams. Rather, the listening task typically requires listeners to follow the elements of the stimulus sequences over the course of each presentation and perceptually group relevant elements sequentially and separate the groups into different running auditory streams. As a result, the objective paradigms can reduce the subject bias associated with the subjective paradigms. Additionally, listeners’ desire of providing highest possible performance in the objective paradigms tend to motivate them to execute at their highest capacity. When a separation-facilitating task is used as an objective approach to study voluntary stream segregation, this motivational aspect would elicit stream segregation ability to its highest level. Considering the aforementioned advantages of an objective paradigm, this study employed a segregation-facilitating task which was modified from the task reported in Nie and Nelson (2015). The direction of focused attention on segregation for better performance in the task resembled the top-down processing for speech perception in background noise where listeners direct their attention selectively to interested speech instead of background noise.

Auditory stream formation has been shown to be dependent on the amount of time the target sequence is presented (e.g., Anstis and Saida, 1985; Cusack et al., 2004). The tendency for segregation to occur increases with longer exposure time to the sound sequence. In other words, auditory stream segregation builds up over time. Using subjective methods, researchers have estimated that stream segregation builds up rapidly over about 10 s, then builds more slowly up to at least 60 s (Moore and Gockel, 2012). While the time course of build-up segregation has not been assessed in CI users using objective methods, our past study (Nie and Nelson, 2015) has shown that the build-up can be observed in a period of 3.1 s for NH listeners with a segregation-facilitating objective paradigm. Nie and Nelson (2015) also revealed that both spectral and AM-rate separations could be cues for the build-up; that is, larger inter-stream separations in either spectrum or AM-rate are associated with greater increases of stream segregation over the course of 3.1 s.

It has been hypothesized that CI users are inferior to NH listeners in stream segregation abilities (e.g., Hong and Turner, 2006; Böckmann-Barthel et al., 2014). Growing evidence has supported this hypothesis for obligatory segregation. For example, using objective segregation-hindering paradigms, some works (e.g., Tejani et al., 2017) have shown that CI users experience weaker perceptual segregation than NH listeners for the same amount of inter-stream frequency separation. Additionally, other works (Cooper and Roberts, 2007, 2009) have argued the absence of obligatory segregation in CI users. Generally, the aforementioned findings suggest that CI users have a lower capacity of perceiving salience of inter-stream differences than NH listeners, which can be attributed to the degradation of auditory signals through cochlear implants. Degraded auditory signals tend to present ambiguous cues for stream segregation which may be modulated by voluntary attention. A few works (e.g., Böckmann-Barthel et al., 2014) have used subjective paradigms to show that CI users are able to form segregated auditory streams even with these ambiguous cues. To our knowledge, only one research group (Paredes-Gallardo et al., 2018a,b,c) has specifically studied voluntary stream segregation in CI users. Using an objective segregation-facilitating paradigm with direct electrical pulse stimuli, this group concluded that CI users were able to voluntarily segregate auditory streams of pulses when the inter-stream difference is in electrode position (Paredes-Gallardo et al., 2018b) or in pulse rate (Paredes-Gallardo et al., 2018c), even when the differences were ambiguous.

These findings suggest that CI users are able to segregate auditory streams based on spectral differences—which is related to the inter-stream electrode-position differences, and temporal-pitch separations—a cue elicited by the inter-stream pulse rate separation. While the direct electrical stimuli in Paredes-Gallardo et al. studies (Paredes-Gallardo et al., 2018b,c) allowed more robust control of stimuli, the acoustic signals and direct electrical stimuli do not activate the electrodes in the identical manner. Thus, the ability to utilize spectral and temporal-pitch cues remains to be examined with acoustic stimuli in CI users. Segregating auditory streams has been suggested to contribute to speech recognition in noise in CI users (Hong and Turner, 2006). A wealth of research has shown that CI users are more vulnerable than NH listeners to distractions when recognizing speech (e.g., Cullington and Zeng, 2008) and investigated various underlying mechanisms for CI users’ higher vulnerability (e.g., Oxenham and Kreft, 2014; Goehring et al., 2021). However, no known research has compared voluntary segregation between CI users and NH listeners to study the mechanism of sequential processing. Examining whether the two groups can attain a comparable level of segregation based on the identical acoustical cues would have implications, from the sequential processing aspect, on understanding CI users’ vulnerability when recognizing speech in noise.

The current study was aimed to evaluate the spectral and temporal-pitch cues for voluntary stream segregation and for the build-up of stream segregation in CI users in comparison to NH listeners. We adopted stimulus constructs and procedures similar to those in Nie and Nelson (2015) with modifications. Specifically, the study was conducted using a segregation-facilitating objective paradigm with stimulus sequences of narrowband noise (NBN) that was amplitude modulated. The stimulus sequences differed in either the frequency region of the NBN or AM-rate for the purpose of examining spectral and temporal-pitch cues for stream segregation, respectively. Each noise band was manipulated such that its bandwidth was constrained within the single excited auditory peripheral filter for NH listeners. For CI users, the bandwidth was restricted to be within the frequency passband of the assigned electrode according to their individual clinical MAP. This manipulation allowed the degrees of inter-stream spectral separation between NH and CI users to be similar for the acoustic stimuli, while limiting the cochlear regions stimulated by the electrical output of the NBN for CI users. However, the inter-stream spectral separations in the internal electrical stimulation for CI users are effectively reduced resulting from the nature that certain noise bands activating other electrodes beside the assigned one (for details, see the electrodogram in Materials and methods). Findings of the current study revealed the effects of reduced salience of internal inter-stream spectral separation on stream segregation.

The aim of studying the build-up stream segregation was motivated by poorly understood listening challenges faced by CI users. For example, whether CI users take a longer time to separate running auditory sequences, which has important implications for CI users’ listening in real life. Studying the build-up stream segregation in both CI and NH groups will allow us to compare how fast the two groups can separate auditory streams of the same acoustic stimuli. Together, the aims of this study include using a segregation-facilitating objective approach to compare voluntary stream segregation abilities with NBN noise in NH listeners and CI users based on inter-stream spectral separations and AM-rate separations, as well as build-up segregation.

## Materials and methods

### Participants

Ten adult listeners between 18 and 69 years of age, three female, seven male, participated in the study. They were divided into two groups: post-lingually deafened cochlear implant (CI) users (6 participants) aged 24–69 years with a mean age of 52.5 years, and normal-hearing (NH) listeners (4 participants) aged 22–60 with a mean age of 37.8 years. All NH listeners had symmetric hearing thresholds no greater than 25 dB HL at audiometric frequencies of 250 to 8,000 Hz, and no greater than a 10 dB difference between ears at the same frequency. All CI users wore only one cochlear implant; if they were bilateral users, they wore the CI on the side perceived to be dominant. All CI participants had no residual hearing, expect one who was a bimodal listener. This bimodal listener did not use their hearing aid in the other ear that was blocked with a foam earplug to avoid the effect of residual acoustic hearing. Table 1 illustrates the demographics of each CI user.

**Table 1:** Participants’ demographics and electrodes to which A and B noise bands were mapped in the moderate and large A-B spectral separations. The center frequencies (CF) of the noise band is also shown.

Participant code	Age (Years)	CI brand	Noise band (B)		Noise band (A)			
					Moderate A–B spectral separation		Large A–B spectral separation	
			Electrode #	CF (Hz)	Electrode #	CF (Hz)	Electrode #	CF (Hz)
CI1	53	Cochlear	11	1808	8	2,927	2	6,418
CI2	69	Cochlear	12	1,683	8	2,871	2	6,485
CI3	69	MED-EL	7	1,632	10	3,064	12	7,352
CI4	43	Cochlear	11	1741	7	3,092	2	6,828
CI5	24	Cochlear	12	1,683	8	2,871	2	6,485
CI6	57	Cochlear	12	1,683	8	2,871	2	6,485

### Apparatus

The stimuli were generated live using a customized MATLAB (R2013a) script at a sampling rate of 44,100 Hz, then processed through a Lynx 22 soundcard installed in a Dell Optiplex 9010 computer, which ran through a DAC1 device. The analog output of the DAC1 was amplified *via* a Tucker Davis Technologies, TDT RZ6 system and presented through a Klipsch RB-51 bookshelf speaker. Stimulus presentation and response recording was controlled by the MATLAB script in conjunction with PsychToolbox (version 3; Brainard, 1997; Pelli, 1997). To record the participants’ responses, an RTbox (Li et al., 2010) was used as the hardware interface. Participants were seated in a sound-attenuated booth at 0^o^ azimuth at a 1-meter distance from the speaker.

### Stimulus sequences

**Figure 1:**
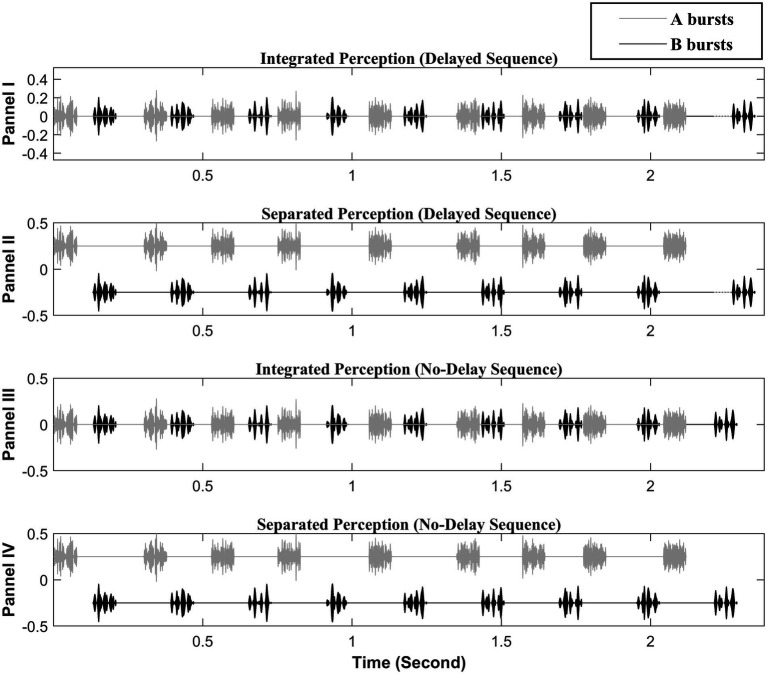
Illustration of the stimulus paradigm (adopted from Nie and Nelson, 2015, with permission). The black segments represent the B bursts that form the attended target subsequence. The light gray segments represent the A bursts that form the unattended distracting subsequence. Panels I and II illustrate the delayed sequences: The dark dotted lines to the left side of the last B burst show the delay of 35 ms for the last B burst. Panels III and IV illustrate the no-delay sequences. The I and III panels depict a visual representation of the integrated perception, while the II and IV panels depict the segregated perception. The large A–B spectral separation is depicted here. The AM rates shown on the A bursts and B bursts are 300 Hz and 50 Hz, respectively. The depicted sequences all consist of 9 pairs of A and B bursts with a duration of 80 ms for each burst. The onset-to-onset time between he first A and B bursts is 130 ms consisting of a silent gap of 50 ms between the offset of A and the onset of B. The B–B onset-to-onset time is 260 ms; in other word, the B–B offset-to-onset time is 180 ms as noted in the text. Note, the last eight A bursts are temporally jittered between the two consecutive B bursts resulting in the offset-to-onset gap between an A burst and an adjacent B burst ranging between 10 and 90 ms.

The stimulus paradigm consisted of sequences of 9 or 3 pairs of A and B noise bursts, in the pattern of ABABAB…. The paradigm is illustrated in Figure 1. The A and B bursts were NBN generated by passing broadband Gaussian noise through 10th order Butterworth filters with center frequencies and bandwidths described in the following paragraph. In some conditions, amplitude modulation was superimposed on A and B bursts. The A and B bursts differed in either center frequency of the noise band, the AM-rate, or both. The duration of each A or B burst was 80 ms including 8 ms rise/fall ramps. A 50-ms silent gap was included between the offset of a burst to the onset of the next with the A bursts (except the initial one in a stimulus sequence) jittering from their nominal temporal location. The amount of jitter was randomly drawn for each jittered A burst from a rectangular distribution between 0 to 40 ms. In other words, the B bursts were presented steadily with a 180-ms gap between the two consecutive ones, except for the last B burst, in any sequence, while the offset-to-onset gap between an A burst and either adjacent B burst ranged between 10 and 90 ms. In some sequences, namely *delayed sequences*, the last B bursts were delayed from their otherwise-steady temporal position by 35 ms; in the other sequences, namely *no-delay sequences*, the last B bursts were temporally advanced by an amount randomly drawn from the rectangular distribution ranging from 0 to 10 ms. As a result, the *delayed sequences* were 2.325 and 0.665 s in duration when the sequences, respectively, consisted of 9 and 3 pairs of A and B bursts, while the *no-delay sequences* were 2.28–2.29 s and 0.62–0.63 s when consisting of 9 and 3 AB pairs, respectively.

The longer sequences were shortened by approximately 1 s from those in Nie and Nelson (2015) to allow the study of build-up within the time course of 3 s. This was designed to address the question raised in Böckmann-Barthel et al. (2014). Using a subjective approach, Böckmann-Barthel et al. examined the time course for CI users to build up stream segregation of sequences of interleaved harmonic tone complexes differed by varied amounts of f0. They noted that the CI users rarely provided first response within 3 s of the stimulus onset to indicate the number of streams they perceived. The authors argued that the initial time point for studying the course of build-up, in turn, should be normalized to the timepoint when the first response was provided. With the approach of normalizing the initial timepoint, the study has shown that CI users may not require time to build up segregation when the f0 difference between the two streams is substantially large. The authors noted that this trend was comparable to NH listeners who showed the no build-up required when segregating streams of harmonic tone complexes which had large frequency differences (Deike et al., 2012). However, Deike et al. (2012) did not undertake the normalized initial timepoint approach and showed that NH listeners reported perceiving segregation within 2–3 s of the onset of a stimulus sequence. Thus, it remained unclear how CI users would compare with NH listeners in build-up segregation within 2–3 s of stimulus onset. A sequence shorter than 3 s with an objective paradigm allowed us to examine this process in the current study.

Three levels of spectral separation between A and B bursts—no-separation, moderate separation, and large separation—were examined. For the NH listeners, by holding the center frequency of B bursts constant at 1,803 Hz, these A–B spectral separations were achieved by setting the center frequency of the A bursts, respectively, at 1,803, 3,022, or 6,665 Hz. As a result, the center frequencies of A and B bands differed by 0.75 and 1.89 octaves for the moderate and large A–B spectral separations, respectively. The three center frequencies coincided with the center frequencies mapped to the 10, 13, and 16th electrodes through typical 16-channel signal processing strategies of the Advanced Bionics technology, thus were selected for NH listeners to simulate separations of assigned channels between A and B bursts around 3 or 6 electrodes for CI users. Noise bursts were set to the narrowest bandwidths allowing a steady presentation level in the sound field in the participant’s location (Walker et al., 1984). Subsequently, bandwidths of 162 Hz were applied for the noise bands centered at 1,803 and 3,022 Hz, and 216 Hz for noise band centered at 6,665 Hz.

**Figure 2:**
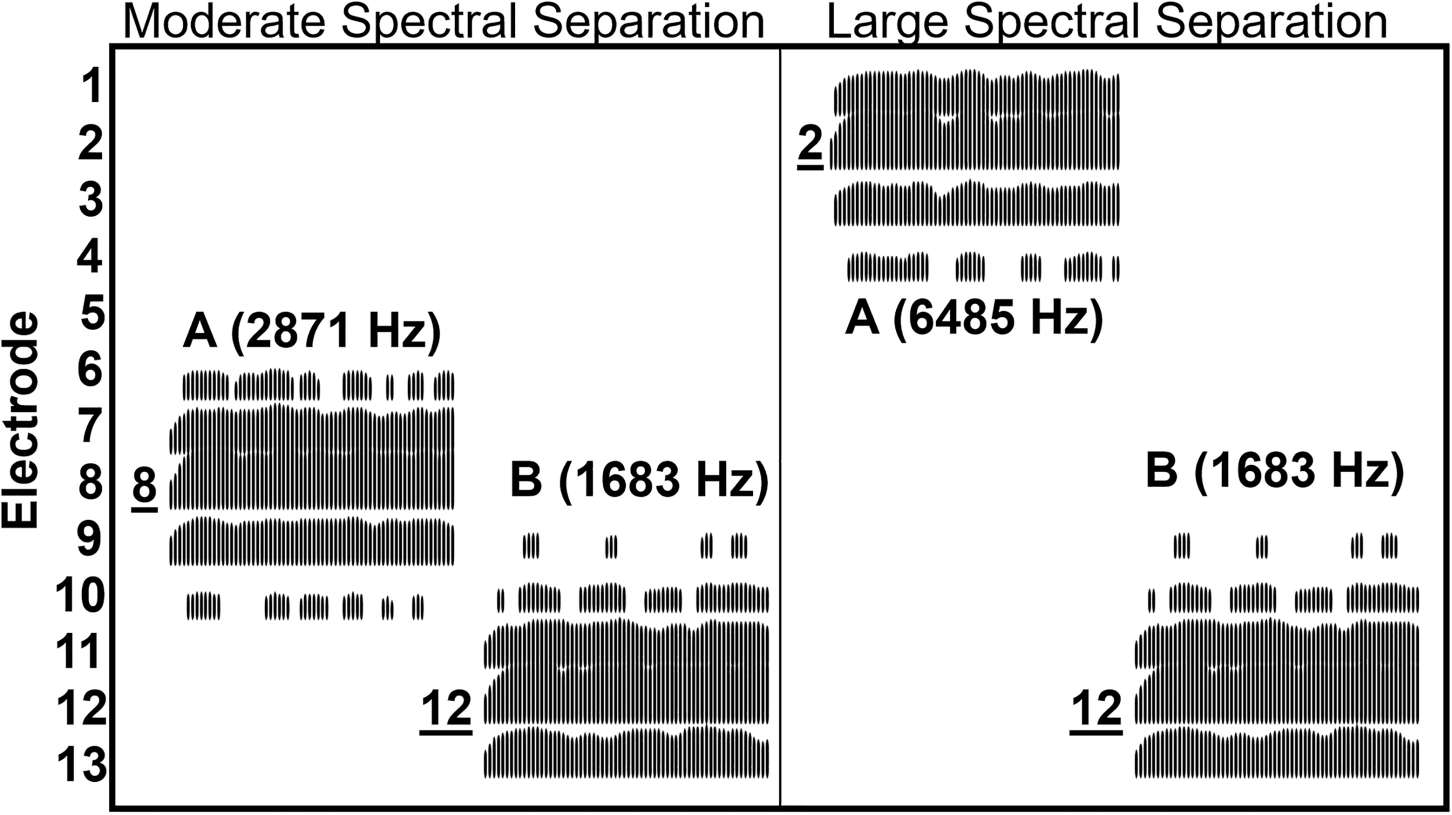
Electrodogram illustrating the electrodes activated by A and B narrowband bursts when they were at the moderate and large spectral separations for the three CI users, for whom the frequencies of stimulus bursts were identical. The electrodogram was generated based on the ACE processing strategy all three CI users used with a default frequency allocation (188–7,938 Hz), stimulation rate (900 Hz), and eight maxima. The A and B bursts are labeled with their corresponding center frequencies in the parentheses. The electrodes are ordered vertically starting from the most basal electrode #1 at the top through electrode #13 at the bottom. Direct labeling with underlined numbers indicate those electrodes to which the A and B bursts were assigned. Note that an A or B burst activated four or five electrodes and did not produce any activation on electrodes #5 and #14–20. The electrodes #14–20 are not shown in the figure.

For the CI users, the center frequency of B bursts was customized for each listener so that it coincided with the center frequency of the signal processing channel in which the 1,803 Hz was allocated in the listener’s clinical MAP. Likewise, the center frequency of the A bursts was customized for each CI users to achieve the three spectral separations. As a result, at the moderate and large A–B spectral separations, the center frequencies of the noise bands separated by 0.70–0.91 octaves and 1.83–2.17 octaves, respectively. Table 1 shows the center frequencies and electrode numbers for the A and B bursts at three levels of A–B spectral separation for each CI user. Through the CI processing, in addition to the assigned electrode, a given noise band effectively activated a number of other electrodes. An electrodogram was computed for the B burst and the two frequency regions of A bursts based on the most common frequency allocation among all CI users. Figure 2 shows the electrodogram demonstrating that a given NBN stimulus activated a total of four or five neighboring electrodes adjacent to the assigned electrode. Specifically, the B burst activated electrodes #9, 10, 11, 12, and 13, while the A bursts activated electrodes #6, 7, 8, 9, and 10 in the moderate spectral separation and electrodes #1, 2, 3, and 4 in the large spectral separation.

The AM-rate alternatives for A bursts were 0 (i.e., no amplitude modulation), 200, or 300 Hz. B bursts were presented either at an AM-rate of 0 or 50 Hz. Three possible AM-rate separations between A and B bursts included no separation wherein both bursts were not modulated (AM0-0), 2-octave separation with A bursts modulated at 200 Hz and B bursts at 50 Hz (AM200-50), and 2.59-octave separation with A bursts modulated at 300 Hz and B bursts at 50 Hz. The depth of AM was 100%.

### Procedure

To account for perceived loudness differences in presentation of frequency-varied stimuli, each participant performed loudness balancing through an adaptive procedure (Jesteadt, 1980) to begin testing. Through the procedure, the levels of an A burst perceived to be equally loud as 60 dB SPL for a B burst were derived separately for the moderate and large spectral separations.

To measure stream segregation abilities based on listeners’ behavioral responses, a single interval yes/no procedure was adopted. On each trial, either a delayed sequence or a no-delay sequence was presented. In a sequence, each B burst was presented at 60 dB SPL and each A burst at the level derived in the loudness balancing procedure. The task was to determine whether the sequence was delayed (i.e., the signal sequence) or no-delay (i.e., the reference sequence). Two graphic boxes on a computer screen, one showing “1 Longer” and one showing “2 Shorter,” respectively, for the delayed and no-delay sequences. The participants responded by pressing number 1 or 2 on the RTbox (Li et al., 2010) to indicate their identification of delayed or no-delay sequence. Feedback was provided following each response by illuminating the box corresponding to the correct answer on the screen. Participants were allowed to take as much time as they needed to make the selection for each trial.

Two blocks of 65 trials were run for each condition with a 50% chance of signal sequences. The first 5 trials served to familiarize participants with the task. The last 60 trials were used to compute the hit rate and false alarm rate from both of which participants’ sensitivity $d^{\prime }$ to the signal sequence was derived from Equation 1, yielding two $d^{\prime }$ scores per experimental condition.

(1) $$d^{\prime }=Z\left(h\right)-Z\left(f\right)$$

where $Z\left(h\right)$ and $Z\left(f\right)\text{,}$ respectively, represent the Z transforms of hit rate and false alarm rate. Our previous studies (Nie et al., 2014; Nie and Nelson, 2015) have shown that this stimulus paradigm encouraged participants to segregate the A and B subsequences to achieve higher $d^{\prime }$ values. In those studies, the baseline performance for the rhythm-based stream segregation, as described at the end of the Procedure, was estimated with a $d^{\prime }$ value of 1.5 on average with stimulus sequences of 12 pairs of broadband noise bursts.^1^ Before the experimental sessions, each participant completed a number of 40-trial training blocks that reflected the task of all the experimental conditions. To proceed to the experimental sessions, all participants were required to achieve a $d^{\prime }$ score of 1.5 or higher in at least one training block for the condition of large spectral separation without AM-rate separation, which suggested their ability to perform the stream segregation task.

A total of 18 experimental conditions were examined, with two sequence durations (9-pair and 3-pair), three levels of A–B spectral separation (no-separation, moderate separation, and large separation), and three AM-rate separations (AM0-0, AM200-50, and AM300-50). The participants undertook these conditions in a pseudorandomized order, such that duration/spectral separation conditions were randomized first, followed by the random order of the AM-rate separations nested under the duration/frequency separation conditions. The two repetitions of the same condition were conducted in two consecutively blocks.

It is noteworthy that the rhythm embedded in the stimulus sequences has been shown to enable voluntary stream segregation (Devergie et al., 2010; Nie et al., 2014). Thus, the stimulus sequences with no inter-stream spectral separation (i.e., the no-separation condition) and no AM-rate separation (i.e., the AM0-0 condition) effectively served as the control condition that provided the baseline performance in the absence of both spectral and AM-rate separations between the A and B streams.

### Data analysis

The statistical package of IBM SPSS for Windows (Version 27.0) was used for data analysis. With either the Shapiro–Wilk test or graphical inspection of histograms, the $d^{\prime }$ scores were found to have violated the assumption of normal distribution overall or for most of the groupings. Thus, a complex Linear Mixed Effects (LME) model was fitted to the $d^{\prime }$ scores of both listener groups. The $d^{\prime }$ data of each listener group were also fitted with an LME model separately to examine the effects of spectral separation separately for each group. The residuals of these LME models were found normally distributed for most of the groupings. For readability, variables fitted in the LME models are specified in the Results section. When pairwise comparisons were performed, the reported p values have been corrected with the Bonferroni approach to control for the familywise error rate.

## Results

**Figure 3:**
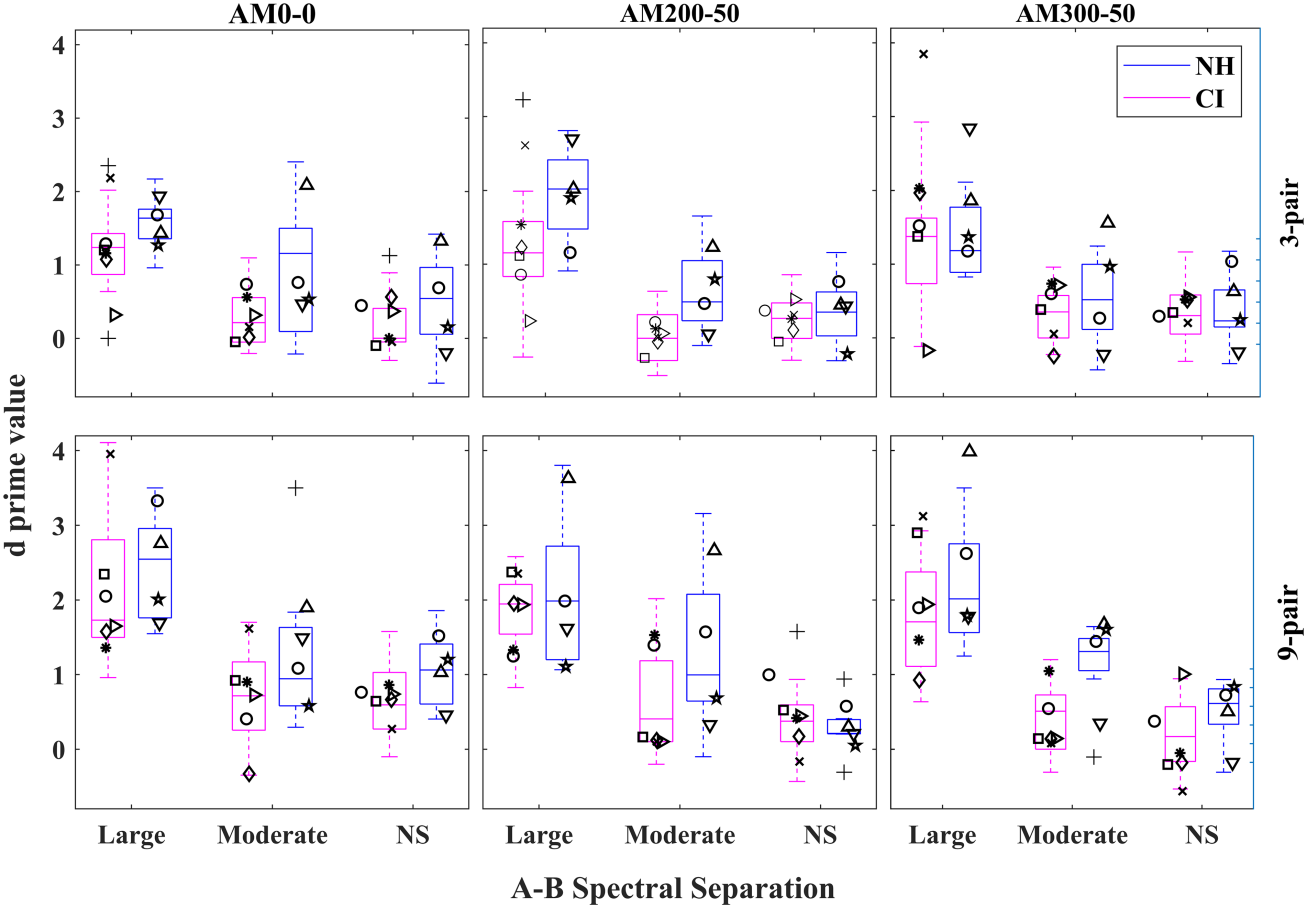
Boxplots illustrating $d^{\prime }$ values for each listener group in different experimental conditions. The boxes show the 25–75th percentile, the error bars show the 5th and 95th percentile, the plus signs show outliers, the solid horizontal line shows median performance. The rows show different sequence durations while the columns show the condition of AM-rate separation. NS = No-Separation. Each participant’s $d^{\prime }$ mean of the two repetitions in each condition is also depicted with a unique symbol for the same listener across all conditions.

Figure 3 depicts the $d^{\prime }$ values for both CI users and NH listeners in different experimental conditions. The $d^{\prime }$ values were higher for the 9-pair than for the 3-pair sequences and highest for the large A–B spectral separation condition out of the three separations. The effectiveness of the listeners training was investigated by comparing the full model with a simpler one. This simpler model excluded the *Repetition* effect but was otherwise identical to the full model with random effects of individual participants and their intercepts.^2^ The simpler model is referred to as the complex model and assessed the fixed-effects factors of *Listener Group (or Group), Sequence Duration (or Duration), Spectral Separation, AM-rate Separation (or AM Separation)*, and all their two-way interactions, two three-way interactions (i.e., *Group* X *Spectral Separation* X *Duration* and *Group* X *AM Separation* X *Duration*).

**Figure 4:**
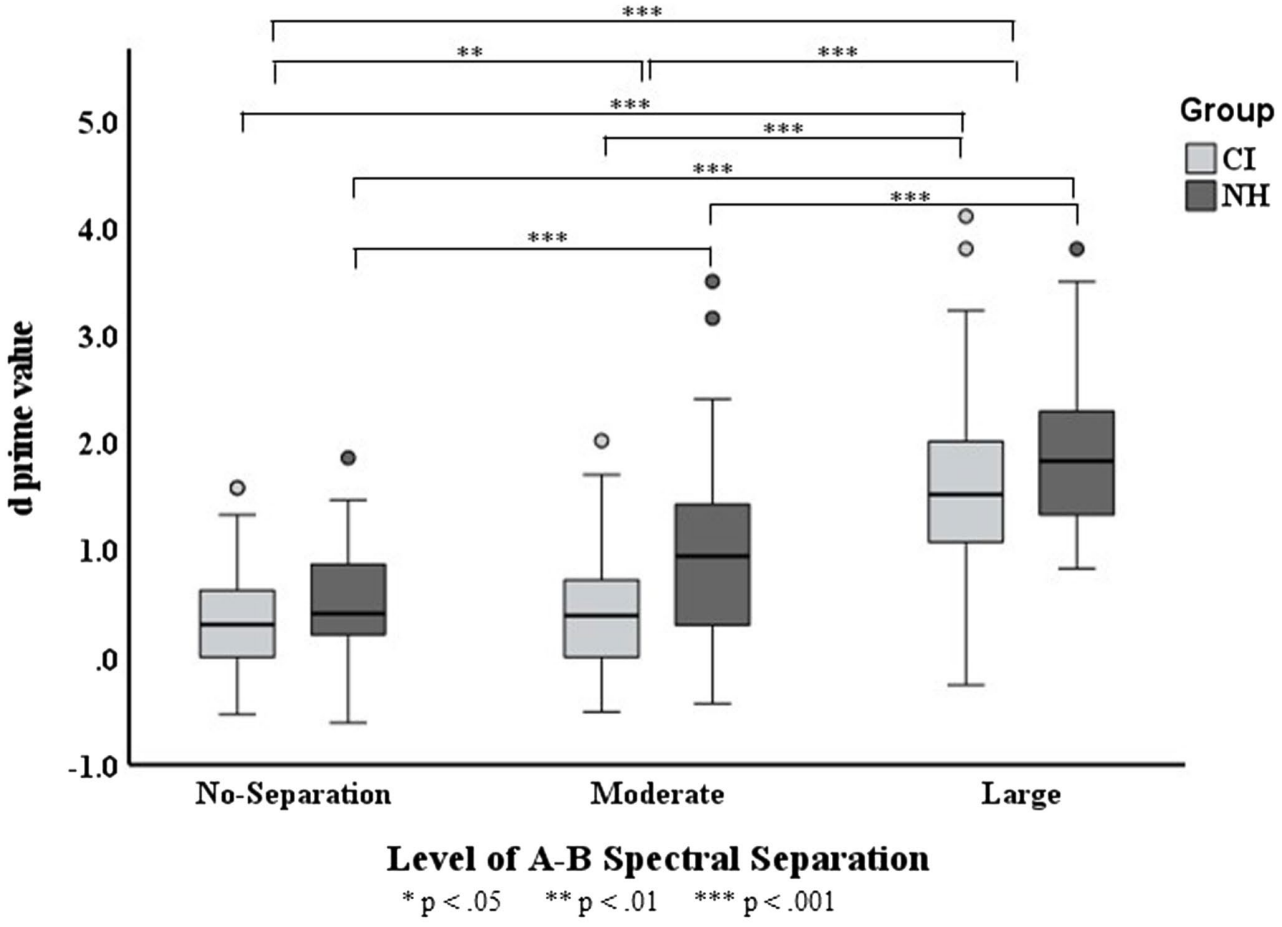
Boxplots for $d^{\prime }$ values across three A–B spectral separations in the CI and NH groups. The boxes show the 25–75th percentile, the error bars show the 5 and 95th percentile, the circle symbols show outliers, the solid horizontal line shows median performance. The statistically significant differences reported in the Results is indicated by asterisks.

**Figure 5:**
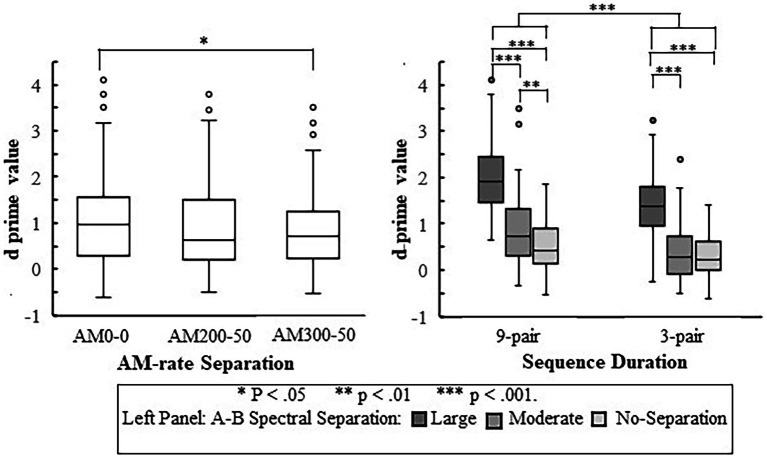
Boxplots for $d^{\prime }$ values in the three AM-rate separations (left panel) and in the two sequence durations at different A–B spectral separations (right panel). The boxes show the 25–75th percentile, the error bars show the 5 and 95th percentile, the circle symbols show outliers, the solid horizontal line shows median performance. The statistically significant differences reported in the Results are indicated by asterisks.

With respect to the main fixed effects, three out of four were found to be significant, *Spectral Separation* [*F*(2, 328) = 165.508, p < 0.001], *AM-rate Separation* [*F*(2, 328) = 4.197, p = 0.016], and *Duration* [*F*(1, 328) = 45.167, p < 0.001]. *Post-hoc* pairwise comparisons revealed that $d^{\prime }$ values progressively increased with the spectral separation, increasing by 0.254 ( p = 0.004) from no-separation to the moderate separation and 1.079 (*p* < 0.001) from the moderate separation to large separation, as illustrated in Figure 4. The main effects of *AM Separation* and *Duration* are shown in Figure 5: The $d^{\prime }$ score was significantly poorer ( p = 0.018) for the largest AM separation (AM300-50) than for the condition with no AM separation (AM0-0) by 0.216; no other pairwise comparisons among the AM separations were found to be significant. In addition, the $d^{\prime }$ mean difference of 0.427 was consistent with higher stream segregation ability with the 9-pair sequences than the 3-pair sequences. The *Group* effect was not significant [*F*(8, 328) = 4.554, *p* = 0.065].

Results revealed that the interaction of *Group* and *Spectral Separation* was significant, *F*(2, 328) = 3.611, p = 0.028. The data of each listener group were fitted with a separate LME model with the fixed-effects and random-effects terms same as those in the full model excluding any term associated with *Group*. As depicted in Figure 4, the $d^{\prime }$ score was found to progressively increase with the spectral separation for NH listeners ( p < 0.001 for any $d^{\prime }$ increase). While the CI users showed the highest $d^{\prime }$ for the large spectral separation ( p < 0.001), their $d^{\prime }$ score did not differ between the moderate- and no-separations ( p > 0.999).

**Figure 6:**
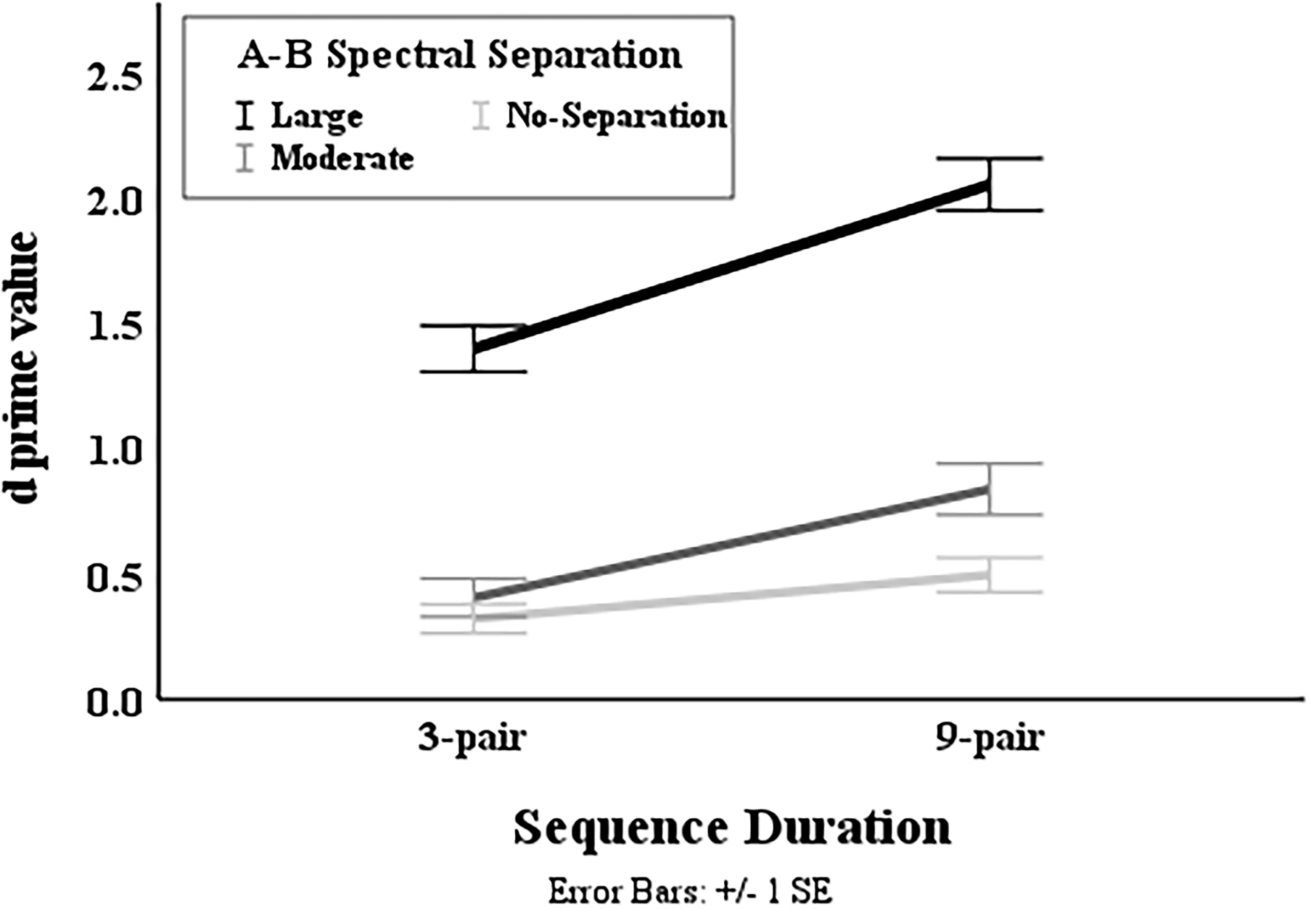
Illustration of mean $d^{\prime }$ value in the two sequence durations at different A–B spectral separations.

Results also revealed a significant interaction in the complex model between *Spectral Separation* and *Duration* [*F*(2, 328) = 4.552, p = 0.011] for both groups. The data of each duration were fitted with a separate LME model with the fixed-effects and random-effects terms same as those in the most complex model excluding any term associated with *Duration*. As depicted in Figure 6, the $d^{\prime }$ value was found to progressively increase with the spectral separation for the 9-pair sequences ( p < 0.003 for any $d^{\prime }$ increase). With the 3-pair sequences, the $d^{\prime }$ score was highest for the large spectral separation ( p < 0.001), but did not differ between the moderate- and no- separations ( p = 0.712).

No significance was found for the interactions of *Duration* X *Group, Duration* X *AM Separation, Group* X *AM Separation, Group* X *Spectral Separation* X *Duration,* and *Group* X *AM Separation* X *Duration*.

## Discussion

With a segregation-facilitating objective paradigm, the current study compared CI users and NH listeners in their ability to voluntarily segregate streams of NBN bursts based on the inter-stream spectral separation or AM-rate separation. The build-up of stream segregation was also investigated and compared between the two groups. The results suggest that CI users are less able than NH listeners to segregate NBN bursts into different auditory streams when they are moderately separated in the spectral domain. Contrary to our hypothesis, results indicated that the AM-rate separation interfered with the ability to segregate NBN sequences for both listener groups. Additionally, our results add evidence to the literature that CI users build up stream segregation at a rate comparable to NH listeners within around 2–3 s of stimulus onset.

### Stream segregation based on spectral separation and AM-rate separation between groups

Consistent with the literature (e.g., Nie and Nelson, 2015; Tejani et al., 2017), the spectral separation of noise bands was shown to be a cue for stream segregation in both NH and CI groups as indicated by the increased $d^{\prime }$ values with the increase of A–B spectral separation. The progressively improved $d^{\prime }$ scores in NH listeners indicate their ability to segregate the A and B subsequences into different streams within 2.3 s when the two streams were at least 0.75 octaves apart. Recall that, the amount of moderate A–B spectral separation was 0.75 octaves for the NH listeners. In contrast, the CI users were unable to segregate A and B subsequences that were moderately separated in spectrum (with the separation of 0.70–0.91 octaves) in 2.3 s, even with the facilitation of focused voluntary attentional effort. Although, when the noise bands are largely separated in spectrum by 1.83–2.17 octaves, the CI participants are clearly able to voluntarily segregate noise streams.

Böckmann-Barthel et al. (2014) used acoustic sequences of harmonic tone complexes to investigate the effect of f0 differences across tone-complex sequences on stream segregation. With a subjective paradigm where CI listeners reported the number of streams perceived, the authors found prevalent stream segregation at the 10-semitone (i.e., 0.83 octaves) f0 difference. This differs from our result, which indicates that CI listeners were unable to segregate streams of NBN separated by 0.70–0.91 octaves. The discrepancy may in part be due to the slower stimulus rate in the current study than in Böckmann-Barthel et al.: In the current study, the stimulus rate was 3.8 AB pairs per second, whereas Böckmann-Barthel et al. repeatedly presented harmonic tone complexes in the ABAB group at a rate of 6 Hz, resulting a stimulus rate of 12 AB pairs per second. The slower rate in the current study may have led to weaker stream segregation (van Noorden, 1975). The discrepancy may also be attributed to the longer observational time for participants in Böckmann-Barthel et al. than in the current study. That is, almost all CI users provided their first responses 3 s after the stimulus onset, whereas the observational sequence duration was 2.325 s or shorter for the listeners in the current study. This potential effect of observational time is relevant to the build-up of stream segregation. Future studies using a larger range of varying sequence duration will allow studying the build-up stream segregation based on the cue of moderate spectral separation in CI users.

Note that, while the A and B bursts each were manipulated to be assigned to a single electrode according to a CI user’s frequency allocation, the electrodogram shows that effectively, each acoustic burst activated a group of 4–5 electrodes. This internal spread of activation substantially reduced the A–B spatial separation in the cochlea. As a result, for the moderate spectral separation, contrasting the 4- or 5-electrode separation between A and B bursts according to the frequency allocation, the electrode groups activated by the two bursts are overlapping; for the large spectral separation, the two bursts activated two electrode groups that are four electrodes apart, instead of two single electrodes separated by 9–10 electrodes (for the MED-EL device, it was 5 electrodes apart).

As alluded in Introduction, using acoustic stimuli does not allow precisely controlling the electrode separation of the streams. In contrast, the use of direct electrical stimulation can constrain each stream to discretely activate a single electrode, in turn providing more precise control on the electrode separation. With direct electrical stimulation, (Paredes-Gallardo et al., 2018b) revealed that, on average, a minimum of 2.8-electrode separation is required for CI users to segregate two auditory streams. The patterns of electrode activation by the acoustic stimuli in our study show a similar trend in that CI users have limited ability to form perceptual streams when the internal activations by acoustic streams are not distinctly separated in the cochlea. It is clear that a distinct four-electrode separation is adequate to allow voluntary segregation. Additionally, using acoustic stimuli allows us to relate our finding to the real-world, which highlights that CI listeners experience sequential interference even when acoustic targets and distractors are apart by more than half octaves.

As the significant interaction of *Group* with *Spectral Separation* reveals, NH listeners are better able to use spectral separation as a cue for the formation of auditory streams. This interaction also indicates that the performance differences between NH listeners and CI users are not comparable across the three spectral separations. There were smaller group differences at the two extreme A–B spectral separations—no-separation and large separation than at the moderate segregation. This suggests that the focused attention may help CI users segregate the auditory streams when the physical cues, such as spectral difference in this study, become more salient. Recent electrooculography (EEG) studies have reported the facilitation of focused attention for the processing of both non-speech and speech signals in competing maskers for CI users. For example, presenting CI users with melody-like interleaved target and distractor streams through direct stimulations, Paredes-Gallardo et al. (2018a) studied the N1 wave—the neurophysiological marker for the initial sensory registration of auditory stimuli—separately for the target and distractor streams in two attentional conditions—the attentive condition, wherein the CI users selectively focused attention on the target stream to perform a listening task, and the ignore condition, wherein the CI users’ attention was focused on a silent video instead of the auditory stimulation. The study revealed that N1 was enhanced in the attentive condition compared to the ignore condition for the target (i.e., attended) stream but remained comparable between the two attentional conditions for the distractor (unattended) stream, demonstrating the facilitative effect of focused attention on the response to non-speech streams. Nogueira and Dolhopiatenko (2022) used EEG to decode attention to two concurrent speech streams—target (i.e., attended) stream and competing (i.e., unattended) stream and found that the EEG-indexed attentional difference between the attended and unattended streams positively correlated with the CI users’ perception of the target speech stream, demonstrating the facilitative effect of focused attention on speech perception. Our results suggest that NH listeners may require less focused attention in separating auditory streams, even when spectral separations are small as compared to CI users, who may require both larger spectral separations and focused attention.

When comparing the CI listeners to the NH listeners, they showed overall comparable $d^{\prime }$ scores. This is likely due to the small sample size which has lowered the statistical power. To examine this proposition, we performed a power analysis which, at the α level of 0.05 and the power of 80% (see the Limitations section for details), estimated the sample size to double in both groups to reveal a significantly higher $d^{\prime }$ for the NH group than for the CI group. Thus, additional participants would be required to further examine the hypothesized lower overall ability to segregate NBN streams for CI users’ than NH listeners.

In contrast to previous results (Nie and Nelson, 2015; Paredes-Gallardo et al., 2018c) that temporal-pitch separations (generated by differences in AM-rate or pulse rate) aid stream segregation, the current study showed that the large AM separation (i.e., AM300-50) significantly interfered with performance with no AM separation (AM0-0). That is, as AM-rate differences increased, performance decreased. In other words, increased AM-rate differences caused stream segregation abilities to decrease. Considering the process of a listener performing the task, this result suggests that the A stream interferes more strongly with participants’ ability to follow the steady B stream when the A and B bursts were amplitude modulated at 300 and 50 Hz, respectively, than when the two bursts were both unmodulated. In Nie and Nelson (2015), wideband noise carriers were used, whereas the current study used NBN carriers. In line with the lower sensitivity in detecting AM with noise carriers of narrower bandwidth than those with wider bandwidth (e.g., Viemeister, 1979; Eddins, 1993), Lemańska et al. (2002) reported a significantly worse AM-rate discrimination with NBN carriers than with wideband noise carriers, likely due to the interference of larger intrinsic amplitude fluctuations of the NBN on the amplitude changes resulting from the amplitude modulation. As a result, the A-to-B perceptual difference elicited by the reduced sensitivity to AM may be insufficient for stream segregation. On the contrary, the AM-elicited A-to-B perceptual difference increased the interfering effect of A stream on the B stream. This may be attributed to the other stimulus difference between the current study and previous works (Nie and Nelson, 2015; Paredes-Gallardo et al., 2018c) in that the slower AM-rate (i.e., 50 Hz) was presented in the attended stream (i.e., B stream) in the current study. In contrast, the faster rate (e.g., 300 Hz for AM-rate or 300 pulses per second) was in the attended stream in the other two works. The sensitivity to AM has been evidenced to depend on the observational intervals (e.g., Viemeister, 1979; Lee and Bacon, 1997) such that, based on the multiple-looks theory (Viemeister and Wakefield, 1991), as the number of “looks” (commonly regarded as equivalent to the cycles) of AM increase, the sensitivity increases. With the 80-ms duration for each stimulus burst in the current study, the number of “looks” for 50-Hz AM was four which was substantially less than the 24 “looks” for the 300-Hz AM, which may affect the equivalence of perceptual salience between the amplitude modulated A and B streams. Future studies should confirm or equate the perceptual salience elicited by different AM rates or pulse rates to examine the effect of temporal pitch on stream segregation.

### Build-up stream segregation in cochlear implant users compared to normal-hearing listeners

Both CI users and NH listeners showed evidence of build-up stream segregation as performance improved with the 9-pair condition relative to the 3-pair condition. Nie and Nelson (2015) used 12-pair sequences to elicit build-up, whereas the current study used 9-pair sequences. This study has shown that even 9-pair sequences are adequately long to elicit build-up stream segregation when compared to 3-pair sequences. That is, over a course of approximately 2.3 s, both NH listeners and CI users increased their performance in segregating the A and B streams with the facilitation of voluntary attention. These results also address the question raised in Böckmann-Barthel et al. (2014) on the build-up stream segregation of CI users within approximately 3 s of the stimulus onset. Recall, in that study where listeners were asked to report the number of streams perceived throughout the course an acoustic sequence of 30 s, the majority of the CI users did not provide their first response until 3 s post the onset of a sequence, leaving the build-up effect uncertain in the short post-onset period. Here, results show that the CI users in our study made use of the short duration of 2.3 s to build up stronger stream segregation for the levels of inter-stream differences specific to this study. These results also suggest that the current stimulus paradigm may be utilized to objectively study CI users’ perception in the earlier period of a listening course in which subjective responses are not readily provided by the listeners.

Our results did not show an interaction between listener group and the duration of the sequence. While this indicates that both participant groups build up stream segregation at comparable rates, the smaller sample size in each group could also be a potential factor. The statistical power of 8.50% estimated in the Limitations section is markedly small, suggesting the likelihood of the non-significant interaction arising from the small sample size is low.

It should note that, the build-up effect revealed in the NH group, at the inter-stream spectral separation of 0.75 octaves or greater in the current study, is not consistent with the Deike et al. (2012) study in which listeners were asked to report the number of streams perceived throughout a course when listening to a sequence composed of two alternating harmonic tone complexes. At the inter-stream f0 separations of 8 semitones (i.e., 0.67 octave) or greater, the likelihood for NH listeners in the Deike et al. study to perceive two separate auditory streams started at the highest level for the first responses (within 1 s of the sequence onset) and remained constant over the entire course of the sequence. In other words, NH listeners did not require time to build up stream segregation in these f0 separations in Deike et al. The faster stimulus rate in the Deike et al., which was 24 AB pairs--the same as in Böckmann-Barthel et al. (2014), may have resulted in stronger segregation which require limited build-up.

### Limitations

The main limitation of this study is the small sample size, which raised a question about the adequacy of power for the statistical analyses. To assess this limitation, using an R package SIMR (Green et al., 2016; Green and MacLeod, 2016), we conducted post-hoc power analyses.^3^ The power analyses started with fitting the $d^{\prime }$ values in the R package LME4 (Bates et al., 2015) through the comprehensive LME model with the random and fixed effects as reported in Results. Note that the two analysis software programs—R and SPSS revealed comparable statistics. The power of a given main fixed effect was estimated in SIMR through the function “powerSim” based on 1,000 simulations applying the “anova” test method. At the α level of 0.05, the estimated power values were greater than 80% for the effects of *Duration, Spectral Separation,* and their interaction (*Spectral Separation X Duration*), 75.8% for *AM-rate Separation*, and 67.30% for the interaction of *Group* X *Spectral Separation*. The non-significant difference between the NH and CI groups was against our hypothesis. The estimated power was 46.90%, suggesting inadequate sample size. Thus, the sample size required for both the CI and NH groups was estimated based on a minimum of the conventionally desired power of 80% (Cohen, 1977, Chapter 2). Following the procedure described by Green and MacLeod, this sample size was estimated to be 12 for each group with a total of 24 participants. To assess the potential contribution of the small sample size to the non-significant *Group X Duration* interaction, the statistical power was also estimated to be 8.50%, suggesting the likelihood of the non-significant interaction arising from the small sample size is low. The above power analysis outcomes suggest that most of the significant effects were fair, but non-significant effect of listener group is likely due to the small sample size.

Additionally, it should note that a participant may perform the task based on an alternative mechanism not involving stream segregation; that is, based solely on detecting the gap between the last A and B bursts, instead of following throughout the entire course of a sequence. The $d^{\prime }$ value in the 3-pair condition with AM0-0 and no A–B spectral separation may approximate the sensitivity to the signal sequences based on this mechanism of gap discrimination. In this condition, the listeners’ rhythm-based stream segregation was limited, if not none, as a result of markedly low observational intervals with three pairs of bursts. With the identical A and B bursts, stream segregation based on the dissimilarity between A and B bursts was not possible. Thus, the participants performed the task primarily by discriminating the A–B gaps and the $d^{\prime }$ (mean = 0.32, SD = 0.54) in this condition can be considered the baseline sensitivity through this mechanism. Participants achieved $d^{\prime }$ values substantially higher than this baseline, in conditions with robust cues for stream segregation, such as the 9-pair conditions (mean = 1.73, SD = 0.83) and the large spectral separations (mean = 1.13, SD = 0.98), which suggests that the mechanism of gap discrimination contributed to the task performance to a modest extent.

### Summary

In summary, NH listeners were able to separate two NBN streams when their spectral separation was moderate or large within the given conditions. In contrast, CI users appeared only to be able to segregate these streams when their spectral separation was large. Additionally, the significant effect of sequence duration in both groups indicates listeners made more improvement with lengthening the duration of stimulus sequences, supporting the build-up effect within the course of approximately 2.3 s. The results of this study suggest that CI users are less able than NH listeners to segregate NBN bursts into different auditory streams when they are moderately separated in the spectral domain. Contrary to our hypothesis, our results indicate that AM-rate separation somewhat may interfere segregation of streams of NBN. Additionally, our results extend previous findings that cochlear implant users do show evidence for the build-up of stream segregation, which appeared to be comparable to NH listeners.

## Data availability statement

The raw data supporting the conclusions of this article will be made available by the authors upon reasonable request, without undue reservation.

## Ethics statement

The studies involving human participants were reviewed and approved by the Institutional Review Board at James Madison University. The patients/participants provided their written informed consent to participate in this study.

## Author contributions

YN conceptualized the study. YN, AM, and HW designed the study. AM and HW collected the data. AM and YN conducted the data analysis. AM, YN, and HW contributed to the writing of the manuscript. All authors contributed to the article and approved the submitted version.

## Funding

This work was supported, in part, by the CHBS Research Grant, CSD Faculty Fund and Ruth Memorial Student Research Grant from James Madison University.

## Conflict of interest

The authors declare that the research was conducted in the absence of any commercial or financial relationships that could be construed as a potential conflict of interest.

## Publisher’s note

All claims expressed in this article are solely those of the authors and do not necessarily represent those of their affiliated organizations, or those of the publisher, the editors and the reviewers. Any product that may be evaluated in this article, or claim that may be made by its manufacturer, is not guaranteed or endorsed by the publisher.

## Footnotes

^1^The rhythm-based baseline was anticipated to be lower than 1.5 due to the shorter sequence of 9 pairs AB bursts versus the 12 pairs in our previous reports.

^2^The effect of *Repetition* was not significant in the full model, indicating limited learning in the experimental session.

^3^*A priori* sample size had been estimated using G*Power (Faul et al., 2009) based on the repeated measures analysis of variance instead of the LME model that was used in the current study. For a medium effect size in Cohen’s f of 0.25, the sample size was 8 in total for the within factors, 2 groups (CI and NH), 36 measures (18 experimental conditions × 2 repetitions), alpha of 0.05, and power of 80%. With respect to the between factors, 28 total participants were predicted based on the same aforementioned statistical parameters, except a large effect size in Cohen’s f of 0.4 was adopted here. The use of a large effect size for the between factors was assumed based on the findings of substantially worse discrimination of acoustic contours based on frequency differences, such as melodic contour discrimination or prosodic discrimination.
